# Clinical applications of machine learning in the survival prediction and classification of sepsis: coagulation and heparin usage matter

**DOI:** 10.1186/s12967-022-03469-6

**Published:** 2022-06-11

**Authors:** Fei Guo, Xishun Zhu, Zhiheng Wu, Li Zhu, Jianhua Wu, Fan Zhang

**Affiliations:** 1 Ningbo Institute for Medicine & Biomedical Engineering Combined Innovation, Ningbo Medical Treatment Centre Lihuili Hospital, Ningbo University, Ningbo, 315040 Zhejiang China; 2 School of Mechatronics Engineering, Nanchang University, Nanchang, 330031 Jiangxi China; 3 School of Information Engineering, Nanchang University, Nanchang, 330031 Jiangxi China; 4 Department of Critical Care Medicine, Qilu Hospital, Cheeloo College of Medicine, Shandong University, Jinan, 250012 Shandong China

**Keywords:** Sepsis, Deep learning, Survival prediction, Convolution neural network, Coagulation

## Abstract

**Background:**

Sepsis is a life-threatening syndrome eliciting highly heterogeneous host responses. Current prognostic evaluation methods used in clinical practice are characterized by an inadequate effectiveness in predicting sepsis mortality. Rapid identification of patients with high mortality risk is urgently needed. The phenotyping of patients will assistant invaluably in tailoring treatments.

**Methods:**

Machine learning and deep learning technology are used to characterize the patients’ phenotype and determine the sepsis severity. The database used in this study is MIMIC-III and MIMIC-IV (‘Medical information Mart for intensive care’) which is a large, public, and freely available database. The K-means clustering is used to classify the sepsis phenotype. Convolutional neural network (CNN) was used to predict the 28-day survival rate based on 35 blood test variables of the sepsis patients, whereas a double coefficient quadratic multivariate fitting function (DCQMFF) is utilized to predict the 28-day survival rate with only 11 features of sepsis patients.

**Results:**

The patients were grouped into four clusters with a clear survival nomogram. The first cluster (C_1) was characterized by low white blood cell count, low neutrophil, and the highest lymphocyte proportion. C_2 obtained the lowest Sequential Organ Failure Assessment (SOFA) score and the highest survival rate. C_3 was characterized by significantly prolonged PTT, high SIC, and a higher proportion of patients using heparin than the patients in other clusters. The early mortality rate of patients in C_3 was high but with a better long-term survival rate than that in C_4. C_4 contained septic coagulation patients with the worst prognosis, characterized by slightly prolonged partial thromboplastin time (PTT), significantly prolonged prothrombin time (PT), and high septic coagulation disease score (SIC). The survival rate prediction accuracy of CNN and DCQMFF models reached 92% and 82%, respectively. The models were tested on an external dataset (MIMIC-IV) and achieved good performance. A DCQMFF-based application platform was established for fast prediction of the 28-day survival rate.

**Conclusion:**

CNN and DCQMFF accurately predicted the sepsis patients’ survival, while K-means successfully identified the phenotype groups. The distinct phenotypes associated with survival, and significant features correlated with mortality were identified. The findings suggest that sepsis patients with abnormal coagulation had poor outcomes, abnormal coagulation increase mortality during sepsis. The anticoagulation effects of appropriate heparin sodium treatment may improve extensive micro thrombosis-caused organ failure.

**Supplementary Information:**

The online version contains supplementary material available at 10.1186/s12967-022-03469-6.

## Introduction

According to the Global Burden of Diseases, Injuries, and Risk Factors Study published in 2020 [1], sepsis is one of the leading causes of morbidity and mortality worldwide. In 2017, the age-standardized mortality due to sepsis equaled 148.1 deaths per 100,000 population [1]. The number of patients with sepsis is estimated to be between 18 and 31.5 million per year, and the mortality is as high as 20% [2–5]. Due to sepsis’ high heterogeneity and complexity [6–8], its unified treatments are impractical. A delay in treatment initiation and support measures increases the mortality of sepsis patients [9–11]. Therefore, enabling physicians to forecast the survival, classify, and characterize sepsis victims in a timely manner is important for obtaining a favorable outcome.

Several prognostic methods in clinical practice have been established as standards for benchmark studies, including APACHE II score [12], SOFA [13], qSOFA, and SIRS [14, 15]. In addition, researchers have been incorporating clinical features such as the dynamic pulse pressure and vasopressor, the delta pulse pressure [16], and the sepsis hospital mortality score [17] into the scoring system to promote quicker and more accurate sepsis diagnosis. However, there is limited evidence of their effectiveness in improving patient outcomes [18].

Machine learning (ML)- based clinical decision support systems for accurate sepsis recognition have received increasing attention in the latest decade [19–22], with many emerging algorithms for prediction [23–35] and classification [36] of the sepsis risk. For example, the existing works utilized Recent Temporal Patterns mining with support vector machine (SVM) classifier[37], congruent cluster analysis [38], K-means clustering method [39], logistic regression, SVM, random forest, decision tree, and extreme gradient boosting machine [40] for sepsis classification or prediction. Chicco et al. [41] used radial SVM, gradient boosting, Naïve Bayes, linear regression, and linear SVM methods to predict the sepsis patients’ survival. Good performance was achieved for positive data instances but poor for negative ones. Although traditional ML algorithms perform well in cluster analysis, the prediction accuracy remains insufficient. As pointed out by Liu and Walkey, more work is required to improve the ML prediction performance [42–44]. Finally, several studies were limited by a lack of external validation and insufficient generalizability.

Various deep learning techniques exhibit excellent learning ability in the existing studies. For example, Kam and Kim [45] trained a long short-term memory and a deep feed-forward network for early detection of sepsis. Scherpf and colleagues [46] proposed a recurrent neural network architecture to predict sepsis using the Medical Information Mart for Intensive Care version 3 (MIMIC-III) dataset. Tom et al.[47] employed a temporal deep learning method to predict the blood culture outcomes in the intensive care unit (ICU). A combination of Convolutional neural network (CNN) features, random forest algorithm, and SOFA score were applied to monitor sepsis patients in [48]. The mentioned studies achieved good performances in disease prediction, but the features’ scale or the number of sepsis cases were relatively small. In particular, the relationships among features were not seriously considered. These limitations are likely to result in overfit and poor generalization.

In the present study, we leveraged the advantages of both deep learning and traditional ML to characterize the sepsis patients’ phenotype. Deep learning models were generated to predict the patients’ survival rate and detect the patients with high mortality. Firstly, the traditional K-means [49] algorithm was used for the distance calculation between the features and for automatic aggregations for the classification of sepsis patients. The optimal number of groups (K) was determined by comparing between the elbow [50, 51] and the silhouette score [52, 53] methods. Principal component analysis (PCA) is used to reduce the dimension of clustering results. The original random vector was transformed by orthogonal transformation to determine the components related or unrelated into a new random vector, and then fewer dimensions were selected. Here, three dimensions are selected to map the futures into three-dimensional space. And then, the survival nomogram was established to determine the significant features with respect to the survival of patients from each phenotype.

A CNN [54, 55] model was selected for its superior representation learning ability. There were two parts in the CNN architecture: a fully connected classifier and the conventional layers. The features extracted by the convolutional layers were classified by the classifier and the efficiency of the classification was ensured by the multi-parameter classifier. The current work established an application platform using only 11 routine blood test variables to enable quick prediction of the 28-day survival rate.

The information from the 11 blood tests (such as the blood cell classification count, blood coagulation function test, arterial blood gas analysis, and liver and kidney function teats) enables physicians from primary hospitals, emergency units, or ICUs to quickly evaluate patients’ risks and tailor the treatments accordingly. The platform was constructed with a multivariate double coefficient fitting function. With the 11 blood test results and the products of any two of the 11 results used as the independent variables, the coefficients of the fitting function were obtained using the full connection network training of deep learning. The proposed method can be helpful even when faced equipment shortage in primary hospitals or emergency units and ICUs or other limitations caused by patient’s condition.

## Methods

**Fig. 1:**
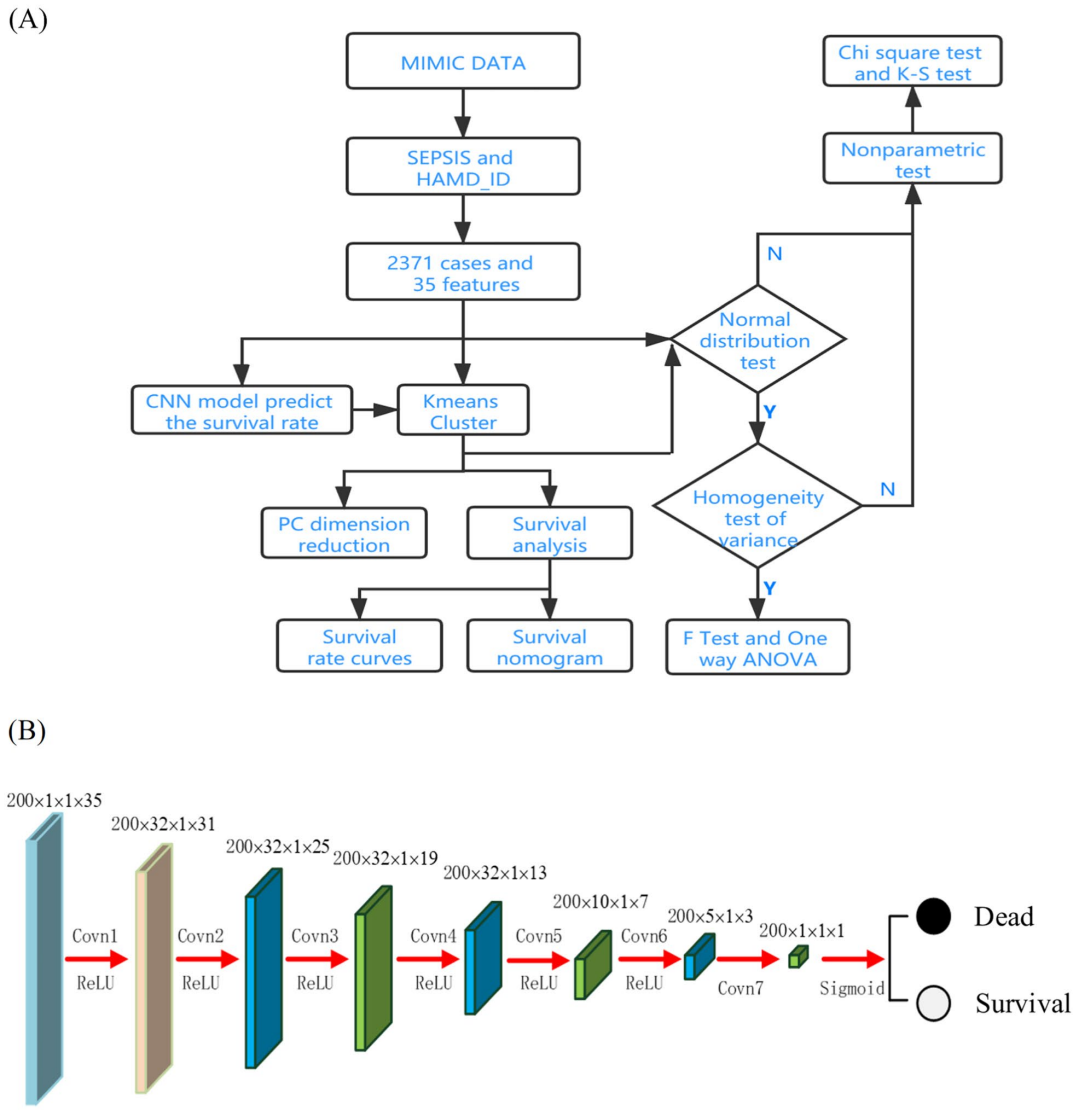
The flow chart of data processing (**A**) and the feature maps of CNN based survival rate (**B**)

This work uses K-means to determine the phenotypes of patients with sepsis, and a deep learning algorithm and a double coefficient quadratic multivariate fitting function (DCQMFF) model to predict the 28-day survival rate of sepsis patients and detect patients with high mortality. The features of the corresponding cases in the phenotype were further analyzed based on the survival prediction results to identify high-risk features leading to death. The flow chart of data processing is shown in Fig. 1A.

## Data collection

This retrospective cohort study was carried out based on MIMIC-III and MIMIC-IV, a large database comprising deidentified health-related data associated with patients who stay in critical care units of the Beth Israel Deaconess Medical Center between 2001–2012 and 2008–2018. The databases include information such as demographics, vital sign measurements made at the bedside (about one data point per hour), laboratory test results, procedures, medications, caregiver notes, imaging reports, and mortality (both in and out of hospital). MIMIC-IV was built upon the success of MIMIC-III and incorporated multiple improvements over its predecessor. Fan Zhang (Record ID 36181465) is certified to get access to the database and is responsible for data extraction. This analysis complied with the Reporting of Studies Conducted Using Observational Routinely Collected Health Data guidelines for administrative claims data [56].

### Statistical analysis

Nonparametric methods were utilized to test the differences in features among subgroups when the data violated the assumptions of normal distribution and homoscedasticity. Two nonparametric tests, Kruskal–Wallis and Jonckheere-Terpstra were both utilized, and the higher *p* value was selected with respect to each comparison. Otherwise, T-test, F-test, and one-way analysis of variance (ANOVA) were conducted accordingly. Additional clinical and laboratory test results are shown in Additional file 1: Table S1 in the Supplemental Material. The *p* values for the association between features and survival were calculated using nonparametric tests on *k*-independent samples.

### K-means clustering of sepsis subgroups and F (PCA)

As an unsupervised ML technique, the K-means clustering method was applied to identify the sepsis clusters in MIMIC datasets [57]. The advantages of this clustering method are its fast speed and less parameters needed. To realize the calculation, the acquired features were taken as direct input and the data were automatically aggregated by the distance calculation. An optimal number of groups (*k*) was determined by compromising between the methods of elbow [58] and the silhouette score [52, 59]. Upon data clustering, PCA was utilized to reduce the data dimensionality to three dimensions to facilitate visualization. Nonparametric tests were used to test the differences among the detected groups.

### Survival rate prediction model based on convolutional neural network (CNN)

Since Hinton and Salakhutdinov [60] proposed a multi-level Boltzmann machine based on a probability graph model in 2006, deep learning has gradually become the leading tool in the field of image processing and computer vision. CNN [54, 61] is one of the prominent deep learning algorithms, with a wide range of applications in various fields and an excellent performance in classification tasks [62]. In addition, advancements in numerical computing equipment further promoted CNN’s representational learning ability.

This work proposes a CNN-based survival rate prediction model to predict sepsis patients’ survival rate. The CNN model contains seven convolutional layers, of which the first six layers use the rectified linear unit (ReLU) as the activation function, and the last one utilizes Sigmoid. Convolution layers extract features extraction, and the activation function adds nonlinear factors. ReLU largely solves the gradient vanishing problem when the model optimizes the deep neural network [63]. The Sigmoid activation function serves to transform the probabilities into the output suitable for binary classification problems. The feature map size for each layer is shown in Fig. 1B.

### Survival rate predication based on a double coefficient quadratic multivariate fitting function (DCQMFF) model

Quadratic fitting function method [64, 65], also known as function simulation or interpolation function method, is recognized as a classical and effective optimization method. To adapt to complex environments, a multidimensional quadratic fitting function [66, 67] was proposed. However, the fitting effect is poor for nonlinear data. To solve this problem, a multivariate quadratic fitting function with double coefficients was proposed to adapt to multi-dimensional nonlinear data for prediction of the survival probability in the current work.

This model considers eleven features, including the most valuable parameters in the SOFA score system that indicate the organ function and two features acquired in Blood Gas Analysis (pH and lactate) critical to estimating the septic shock. More precisely, the considered futures are Creatinine, Hemoglobin, the International standardized ratio of prothrombin time (INR-PT), Lymphocytes, Neutrophils, Platelet Count, Partial Thromboplastin Time (PTT), White Blood Cells, Lactate, Bilirubin, and pH. These features indicate the sepsis severity, with several of them correlating.

First, the data were normalized. To avoid zero minimum values in the normalization process, the formula ${x}^{*}=\frac{x-\mathrm{min}*0.99}{\mathrm{max}-\mathrm{min}}$ is selected. In this formula, *x* is the element before normalization, x^*^ is the normalized element, and max and min are the maximum and minimum values of a feature, respectively. The values of the 11 features are regarded as independent variables ${x}_{i},i\in \{\mathrm{1,2},\dots ,11\}$. Survival and death probabilities are considered as a two-dimensional dependent variable, i.e., *y* = (0,1) or (1,0). The DCQMFF model is defined as:

$${y}_{1}=\sum_{l=1}^{33}{b}_{l}{a}^{l}+\sum_{l=1}^{33}{\sum_{i=1}^{11}{{{b}_{l}a}^{l}}_{i}x}_{i}+\sum_{l=1}^{33}{\sum_{1=i\le j}^{11}{{{b}_{l}a}^{l}}_{ij}}{x_{i}}{x}_{j},$$

$${y}_{2}=\sum_{l=1}^{33}{c}_{l}{d}^{l}+\sum_{l=1}^{33}{\sum_{i=1}^{11}{{{c}_{l}d}^{l}}_{i}x}_{i}+\sum_{l=1}^{33}{\sum_{1=i\le j}^{11}{{{c}_{l}d}^{l}}_{ij}}{x_{i}}{x}_{j},$$

$$y=\left(\frac{{e}^{{y}_{1}}}{{e}^{{y}_{1}}+{e}^{{y}_{2}}},\frac{{e}^{{y}_{2}}}{{e}^{{y}_{1}}+{e}^{{y}_{2}}}\right)$$

where $\frac{{e}^{{y}_{1}}}{{e}^{{y}_{1}}+{e}^{{y}_{2}}},\frac{{e}^{{y}_{2}}}{{e}^{{y}_{1}}+{e}^{{y}_{2}}}$ represents the probability of survival and death, respectively (note, $\frac{{e}^{{y}_{1}}}{{e}^{{y}_{1}}+{e}^{{y}_{2}}}+\frac{{e}^{{y}_{2}}}{{e}^{{y}_{1}}+{e}^{{y}_{2}}}=1$). Double the coefficients can help avoid over fitting caused by a fast dimensionality reduction, thus improving the model’s generalization ability.

The processed data were divided into training data and test data according to a 7:3 ratio. To prevent class imbalance, negative cases were up-sampled by means of replication, random generation according to the median of negative cases features, and adding random noise to the cases to keep the proportion of positive and negative cases nearly equal [68]. Then, the model was trained on the training data and verified using the test data. This procedure processes only the 11 aforementioned features to predict the survival probability. Receiver operating characteristic curve (ROC) [69] was used to evaluate the effectiveness of the CNN and DCQMFF prediction models. The ROC curve is created by plotting the true positive rate (TPR) with respect to the false positive rate (FPR) at various threshold settings and depicts a trade-off between sensitivity and specificity. Thus, the curve summarizes the binary classifier’s performance by combining the confusion matrices at all threshold values. The area under the ROC curve (AUC) measures the classifier’s ability to distinguish between positive and negative classes. The closer the AUC to 1, the better the model at distinguishing the two classes. Finally, accuracy, precision, recall, and *F*_1_-score are four popular metrics for evaluating the performance of classification methods. The CNN and DCQMFF models’ prediction results are compared to those of the random forest, logistic regression, lasso regression, and other methods considered by Chicco et al. [41] (Table 1).

**Table 1:** **|** Comparison of the performance of multiple prediction models

Methods		Accuracy	Precision	Recall	*F*_1_	AUC
Random Forest	Training	0.851	1.000	0.238	0.384	0.619
	Test	0.808	0.909	0.068	0.127	0.533
Logistic Regression	Training	0.825	0.629	0.256	0.364	0.610
	Test	0.808	0.567	0.260	0.357	0.605
Lasso Regression	Training	0.825	0.762	0.148	0.248	0.568
	Test	0.813	0.710	0.151	0.249	0.567
Radial SVM [40]	Training	0.515	0.970	0.491	0.652	0.701
	Test	0.337	0.896	0.204	0.333	0.586
	Val	0.806	0.849	0.920	0.883	0.642
Gradient boosting [40]	Training	0.851	0.934	0.899	0.916	0.690
	test	0.718	0.822	0.816	0.819	0.574
	Val	0.828	0.885	0.905	0.895	0.682
Bayes [40]	Training	0.567	0.965	0.553	0.703	0.649
	Test	0.465	0.861	0.405	0.551	0.562
	Val	0.828	0.891	0.895	0.893	0.713
Linear regression [40]	Training	0.801	0.943	0.835	0.886	0.599
	Test	0.679	0.828	0.763	0.794	0.541
	Val	0.788	0.885	0.842	0.863	0.689
Linear SVM [40]	Training	0.337	0.896	0.205	0.333	0.586
	Test	0.467	0.861	0.407	0.553	0.586
	Val	0.818	0.873	0.906	0.889	0.676
Sofa Score [13] DCQMFF (proposed)	All data	0.752	0.371	0.327	0.348	0.807
	Training	**0.822**	**0.822**	**0.821**	**0.822**	**0.896**
	Test	**0.821**	**0.812**	**0.812**	**0.812**	**0.885**
	Val	**0.775**	**0.764**	**0.754**	**0.759**	**0.849**
CNN (Proposed)	Training	**0.928**	**0.924**	**0.856**	**0.888**	**0.953**
	Test	**0.924**	**0.887**	**0.845**	**0.865**	**0.947**
	Val	**0.834**	**0.825**	**0.818**	**0.821**	**0.909**

### Nomograms

The survival rate curves were calculated, and the statistical analysis of different phenotypes was performed for dataset. Nomograms are prediction models that estimate an individual’s survival by considering multiple clinical variables and their interdependence. Thus, nomograms can provide an overall probability of a specific outcome for an individual patient, offer a more accurate prediction than conventional staging or scoring systems, and, accordingly, improve personalized decision-making in sepsis therapy. The survival nomogram was established using R software by integrating age and other features.

### Hardware environment

The proposed models were run in a hardware environment comprising Intel(R) Core (TM) i5-6200U CPU @2.40 GHz, with 8 GB memory. The workstation’s operating system was Windows 10. The integrated development environment and the deep learning symbolic library were PyCharm-Python 3.8 and PyTorch 1.6.0, respectively.

## Results

### Data description

In MIMIC-III, there were totally 2902 cases of sepsis, 531 sepsis patients were excluded due to the lack of some blood test results. The rest were divided randomly into a training set and a test set at a ratio of 7:3. One thousand six hundred and sixty-one cases were assigned into the training set and 710 into the test set. The 35 blood tests were used in K-means and CNN methods. Length of hospital stay was omitted. In MIMIC-IV, there were 12657 cases of sepsis. To balance the number of dead and surviving cases, more than 3000 cases with the 35 blood test results were extracted and served as validation data set for the two proposed prediction models.

### Identification of the subgroup phenotype and high-risk patients

**Fig. 2:**
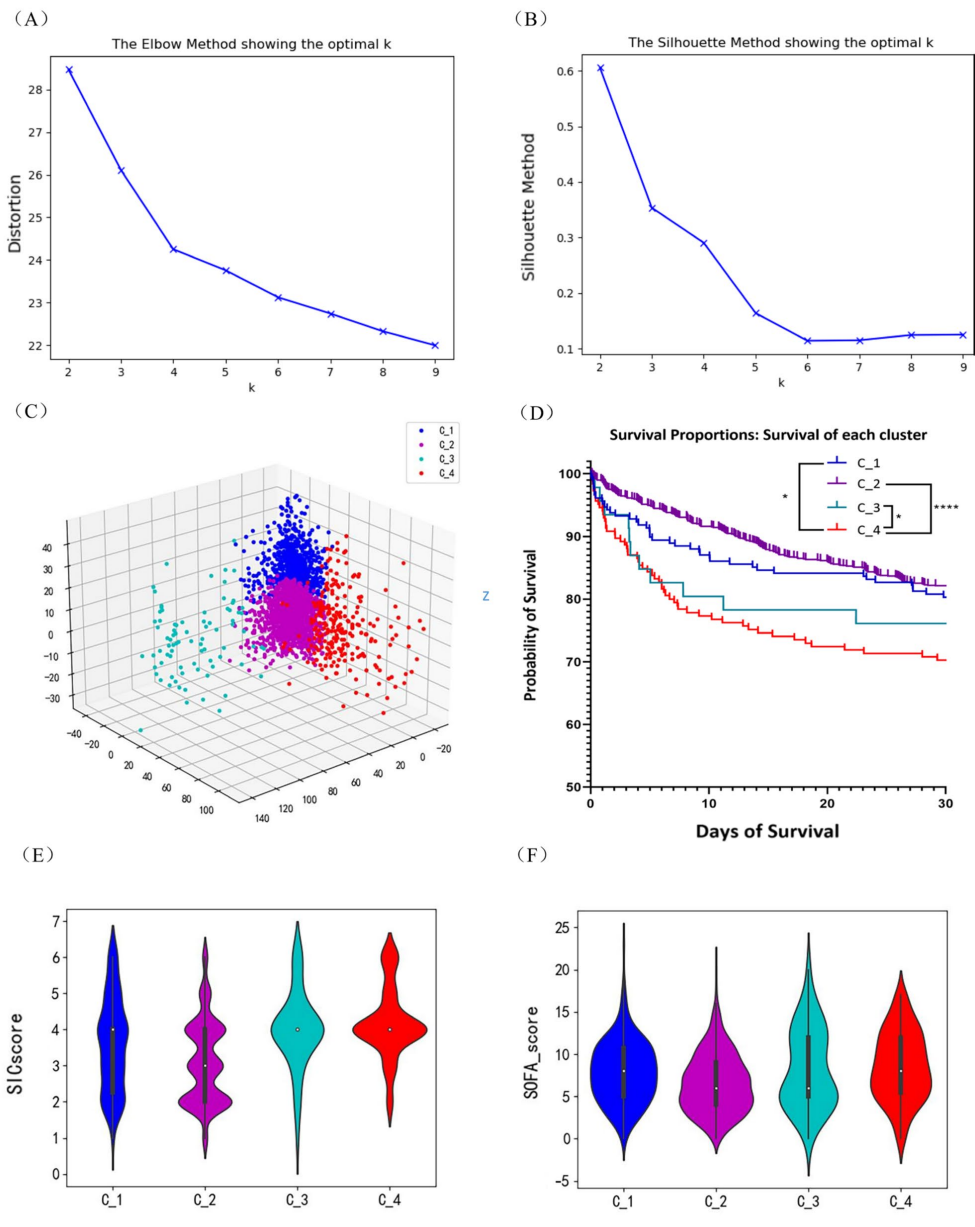
Identification of the subgroup phenotype with *k*-means clustering. The K value was optimized by compromising between the elbow method (**A**) and the silhouette coefficient method (**B**); **C**. The 3D PCA plot visualizes the 4 clusters; **D**. Survival curves; E. SIC score; F. SOFA score. * P < 0.05, ****P < 0.0001 analyzed by log-rank test of Mantel or Gehan-Breslow Wilcoxon test with the higher P value presented

Survival prediction enable detect cases of high mortality probability. However, it is difficult to determine the features which lead to death with the survival prediction tools. Thus, the sepsis population was clustered using K-means clustering, and the obtained groups’ phenotype features were analyzed. The elbow and silhouette score methods (Fig. 2A, B) indicated the presence of four clusters, i.e., K = 4 was selected. Upon the K-means classification, a 3D PCA plot was generated (Fig. 2C). The data set was phenotyped by the K-means method into four clusters including C_1 with 211 cases in the training set and 90 in the test set, C_2 with 1215 and 520, C_3 with 46 and 19, and C_ 4 with 189 and 81.

**Fig. 3:**
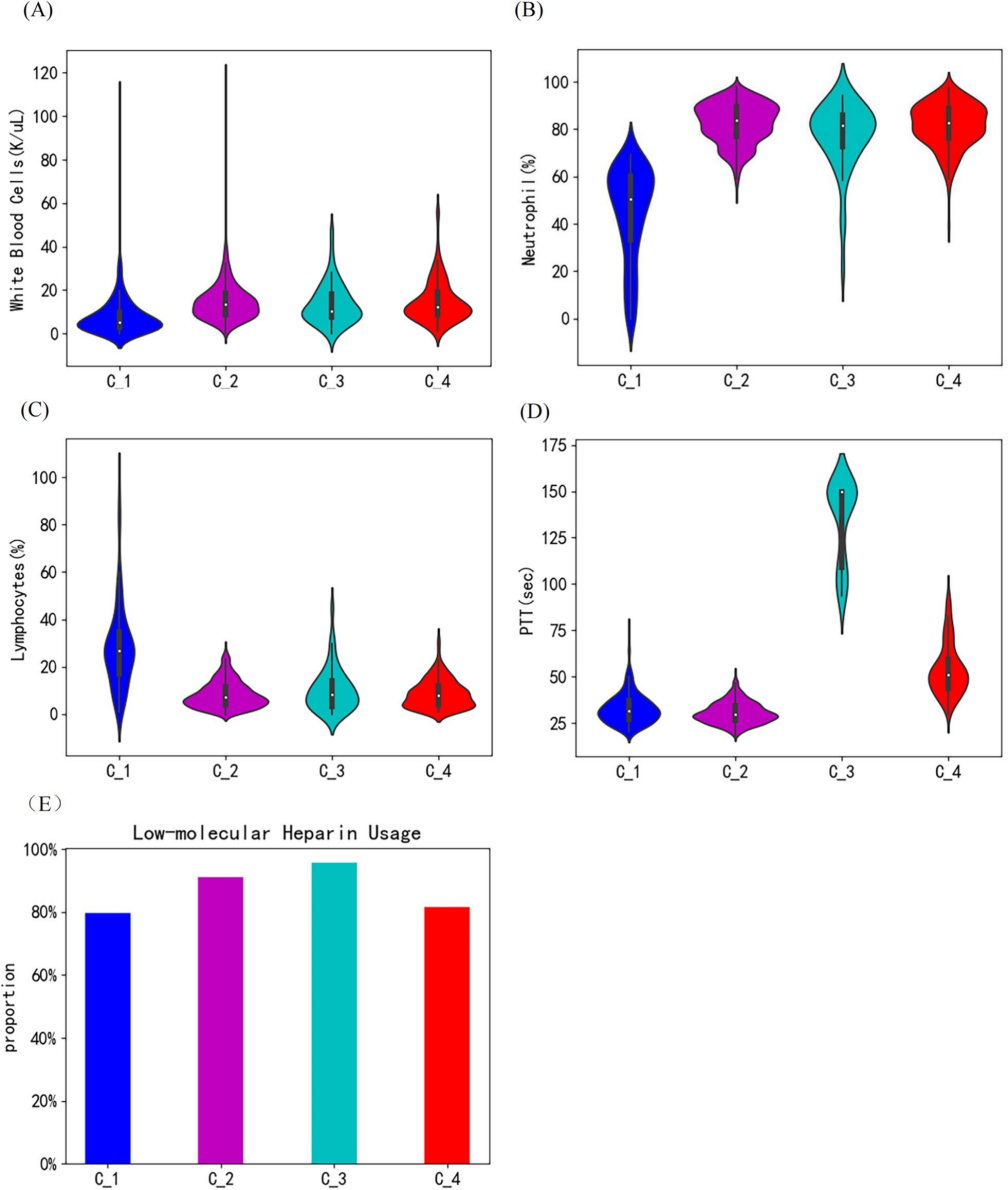
Major features of the four clusters. **A** White blood cell counts (K/µL); **B** Neutrophil proportion (%); **C** Lymphocytes proportion (%); **D** PTT (sec.); **E**. Low-molecular Heparin usage proportion within patients from each cluster (%)

Among the four clusters, Cluster C_2 has the highest survival rate (Fig. 2D). In accordance, this cluster exhibited the lowest SIC and SOFA score (Fig. 2E ,F), further validating the prediction method. C_1 also has a high survival rate. Patients in this cluster are characterized by a low white blood cell count (Fig. 3A) and neutrophil proportion (Fig. 3B) but the highest lymphocyte proportion (Fig. 3C). C_4 is identified as septic patients with abnormal coagulation and had the worse prognosis, characterized by slightly prolonged PTT. C_3 is identified by significantly prolonged PTT (Fig. 3D), high SIC, and higher heparin-using proportion (Fig. 3E) among its patients than those from other clusters. The early mortality rate of patients in C_3 is high but with a better long-term survival rate than those in C_4. Other features of the 4 clusters were plotted in Additional file 1: Figure S1 and S2. The nomograms of each cluster are also established for reference (see Additional file 1: Figure S3).

**Fig. 4:**
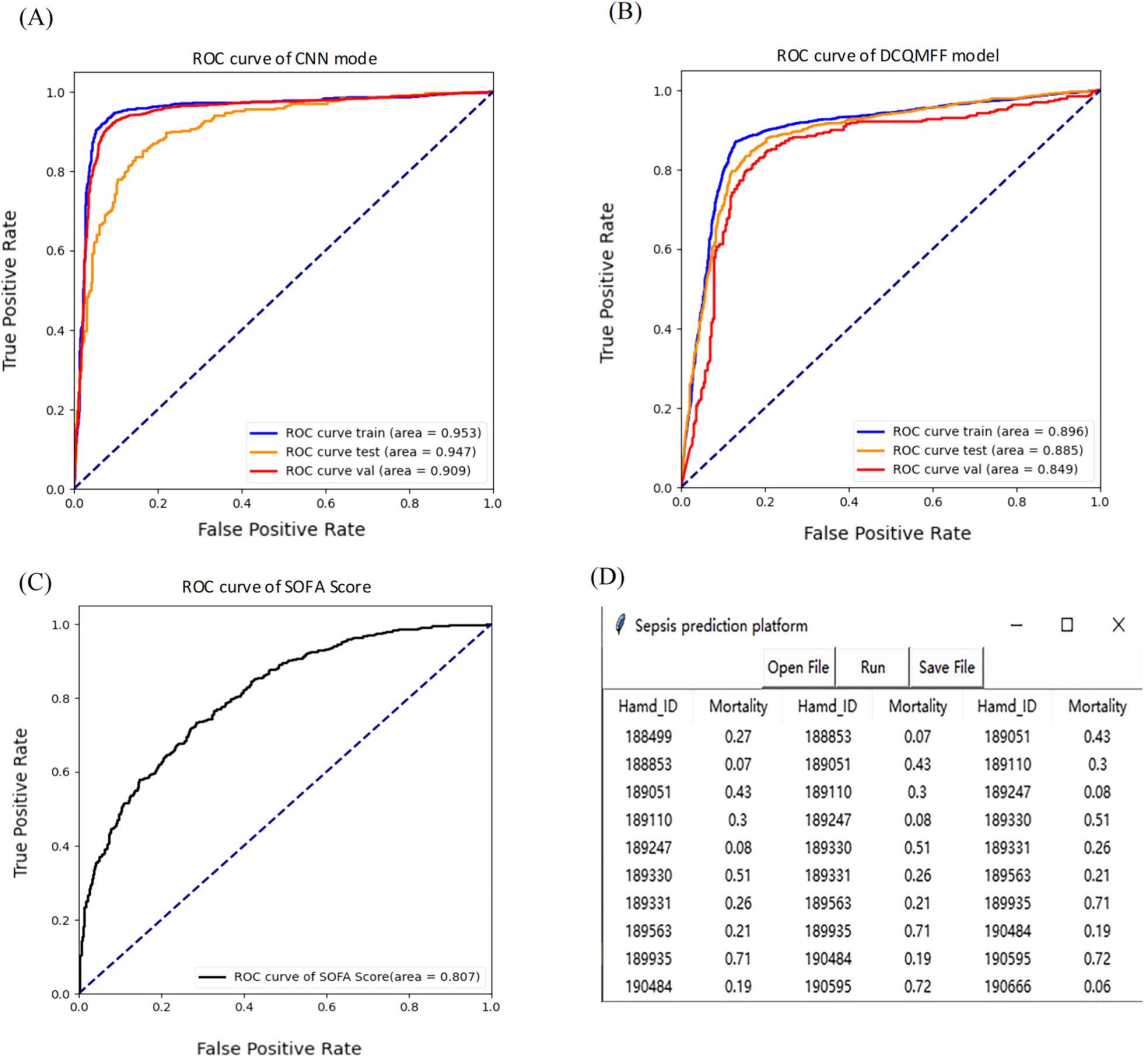
The nomograms predict 28-day survival using 35 clinical features. The nomograms were generated from all the 2371 sepsis cases from MIMIC-III. To use the nomograms, locate patient’s variable on the corresponding axis, draw a line to obtain the point’s axis, sum the points, and draw a line from the total point’s axis to the 28-day survival probability

Table 2, 3 list the features for which the nonparametric tests found the most significant differences in training and test sets respectively. The top ten heterogeneous features shared in the training and test datasets are PTT, neutrophil percentage, PT, INR-PT, lymphocyte percentage, white blood cell count, platelet count, mean corpuscular hemoglobin concentration (MCHC), albumin, and red blood cell count. The survival nomograms were generated for all cases to clarify the relationship between features and death risk using the 35 features and survival information of all 2371 patients in MIMIC-III (Fig. 4).

**Table 2:** The heterogeneous features for training set (1661 cases) according to blood tests

Features	Cluster one	Cluster two	Cluster three	Cluster four	*P* value
number of each cluster	211	1215	46	189	
survival (%)	165 (78.2%)	1005 (82.7%)	35 (76.1%)	132 (69.8%)	0.020
Age, median (IQR), year	66 (54–76)	66 (54–76.5)	67.5 (55–80)	66 (57–77)	0.933
Male, no. (%)	102 (48.3%)	663 (54.6%)	17 (37.0%)	110 (58.2%)	0.023
Top ten blood test varies, median (IQR), unit					
PTT, sec	30.6 (26.5–36.3)	29.6 (26.2–34.3)	150 (122.9–150)	51.6 (43.5–60.6)	0.000
Neutrophils, %	51 (33–61.7)	84 (77–89.9)	81.8 (77.2–86)	83 (76.4–89)	0.000
PT, sec	14.3 (13.1–16.4)	14.2 (13.1–16.0)	17.7 (15.3–23.8)	30.2 (21.9–43.1)	0.000
INR(PT), NULL %	1.3 (1.1–1.5)	1.3 (1.1–1.5)	1.9 (1.5–3.2)	3.4 (2.2–5.2)	0.000
Lymphocytes, %	26 (15.5–34.3)	7 (4–11.3)	7 (4.6–14.0)	8 (4–13)	0.000
White blood cells, K/μL	6.1 (3.4–10.8)	13.2 (8.9–8.1)	10.7 (7.0–8.4)	12.1(8.5–17.4)	0.000
Platelet count, K/μL	2.3 (2.0–2.5)	2.4 (2.2–2.5)	2.3 (2.2–2.4)	2.3 (2.1–2.5)	0.000
MCHC, %	33.3 (32.3–34.3)	33 (32–34.1)	32.5 (32.2–34)	32 (31–33.3)	0.000
Albumin, %	2.9 (2.5–3.3)	2.9 (2.5–3.4)	2.9 (2.5–3.4)	2.9 (2.4–3.2)	0.000
Red blood cells, K/μL	3.5 (3.1–4.0)	3.7 (3.3–4.2)	3.7 (3.2–4.0)	3.7 (3.1–4.0)	0.000

**Table 3:** The heterogeneous features for test data (710 cases) according to blood tests

Features	Cluster one	Cluster two	Cluster three	Cluster four	*P* value
Number of clusters	90	520	19	81	
survival (%)	68 (75.56%)	416 (80%)	13 (68.42%)	57 (70.37%)	0.152
Age, median (IQR), y	64 (51.5–77.5)	66 (54–77)	60 (51.3–69.3)	65 (54–74)	0.364
Male, no. (%)	55 (61.11%)	296 (56.92%)	12 (63.16%)	49 (60.49%)	0.797
Top ten blood test varies, median (IQR), unit					
PTT, sec	31.7 (27.7–36.5)	30.1 (26.6–34.2)	150 (119.0–150)	50.5(43.6–57.7)	0.000
Neutrophils, %	52 (36.3–61.7)	83 (77–88.7)	81 (70.2–87.5)	81 (76.9–88.6)	0.000
PT, sec	14.4 (13.3–17.1)	14.3 (13.1–16.3)	19.5 (15.4–23.1)	28 (19.6–39)	0.000
INR(PT), NULL %	1.3 (1.2–1.7)	1.3 (1.1–1.6)	1.9 (1.6–2.7)	3 (2–4.3)	0.000
Lymphocytes, %	27.1 (18.5–35.3)	7.1 (4–11.9)	9.7 (3.7–11)	7.2 (3–12)	0.000
White blood cells, K/μL	4.7 (2.8–10.3)	13.0 (8.4–18.4)	9.6 (7.4–18.2)	12.4 (8.2–19.2)	0.000
Platelet count, K/μL	2.2 (1.9–2.5)	2.4 (2.2–2.5)	2.4 (2.1–2.6)	2.3 (2.2–2.5)	0.000
MCHC, %	33.4 (32.3–34.6)	33 (32–34)	32.7 (30.5–33.6)	32.5 (31.2–33.6)	0.000
Albumin, %	2.9 (2.6–3.4)	2.9 (2.6–3.4)	2.9 (2.8–2.9)	2.6 (2.3–3)	0.000
Red blood cells, K/μL	3.4 (3.0–4.0)	3.7 (3.3–4.3)	3.8 (3.4–4.0)	3.5 (2.9–3.9)	0.000

### Survival predictions with CNN based and DCQMFF model with external cohort validation

**Fig. 5:**
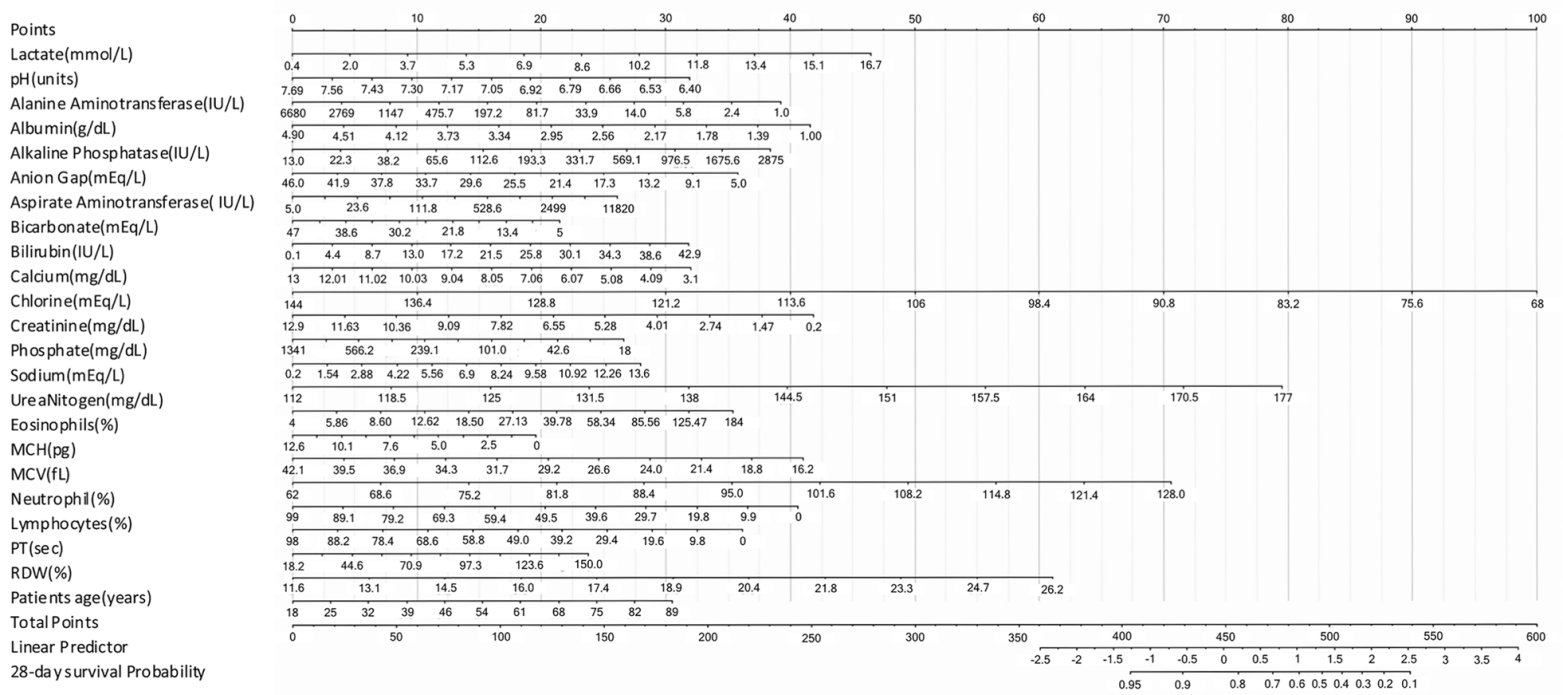
Survival prediction with CNN based and DCQMFF model. ROC curve of the CNN based model (**A**), and DCQMFF model (**B**); **C**. a demo analysis with DCQMFF-based application platform; **D**. ROC curve of the SOFA score

A multivariate approach to predict mortality outcomes of sepsis patients was utilized. The CNN based model was tested using all 35 blood tests, whereas the DCQMFF prediction model used only 11 blood tests. The obtained ROC curves for CNN (Fig. 5A) and DCQMFF (Fig. 5B) models on training, test, and validation sets are shown. The ROC curves for training, test, and validation sets are virtually smooth, suggesting that an overfit is unlikely, the predictive partition analysis verified that the blood tests are strong predictors of sepsis patients’ status. For the CNN model, the AUC scores for the training, test, and validation sets are 0.953, 0.947, and 0.909, respectively. All of the AUCs are close to 1, indicating that the proposed survival prediction model has a good performance in distinguishing the 28-day survivals of sepsis patients. Figure 5B shows the results for the DCQMFF prediction model. While not as good as CNN, DCQMFF performs well using 11 features, with the AUC values for the training, test, and validation sets equal to 0.896, 0.885, and 0.849, respectively. A demo analysis with DCQMFF-based application platform is shown in Fig. 5C.

The traditional survival prediction methods utilized the same 35 features, and the obtained AUC values for the test data are 0.533, 0.604, and 0.567 for Random Forest, Logistic Regression, and Lasso, respectively. The SOFA score is generally applied in the ICU to access multi-organ dysfunction or failure, which is calculated based on PaO2/FiO2, platelets count, bilirubin level, cardiovascular hypotension, Glasgow Coma Scale (GCS), and creatinine level. When the SOFA score exceeds 12, the mortality surpasses 50% [13]. As shown in Fig. 5D, the prediction SOFA score reached an AUC of 0.807, with the lower and upper bounds equal to 0.783 and 0.823, respectively.

Next, the survival prediction performance of the methods proposed by Chicco et al. [41] with age, sex, and septic episode number alone was compared using MIMIC-III as training and test data and MIMIC-IV as validation data. The AUC values for the test data are 0.586, 0.574, 0.562, 0.541, 0.586 and for the validation data 0.642, 0.574, 0.713, 0.689, 0.676 for radial SVM, Gradient boosting, Bayes, Linear regression, and Linear SVM methods, respectively. Although the proposed methods achieved good results regarding major indicators, the true negative rate (TNR) and AUC were low. This issue was likely caused by the class imbalance. Both the CNN and DCQMFF models exhibited outperformed these methods, with an accuracy of 83.37% and AUC of 0.908 for the CNN model and an accuracy of 77.5% and AUC of 0.849 for the DCQMFF on the validation data (Table 1).

## Discussions

Due to the sepsis patients’ heterogeneity and the need to understand features leading to death, this work clustered the sepsis populations and studied the phenotypes. The sepsis patients were divided into four groups according to the Elbow and silhouette score methods analysis on the MIMIC-III datasets. Obtained groups differed in their survival rates, and the phenotypes leading to certain outcomes were analyzed.

Besides, each group was further characterized, and patients in C_4 were detected as those who had complicated septic coagulopathy and a significantly prolonged PT time. These findings indicate that sepsis patients with coagulation disorder are often faced with a poor outcome, which agrees with previous studies [70, 71]. However, patients grouped in C_3 had high early mortality, which can be related to extended prolongation of early PTT. In addition, the proportion of heparin sodium usage in this cluster was significantly higher than in other clusters. However, the long-term survival rate of C_3 patients was significantly better than that of C_4, suggesting anticoagulation effects of heparin sodium improved organ failure caused by extensive micro thrombosis [72, 73] and that abnormal coagulation resulting in micro thromboembolism can aggravate organ failure and increase mortality during sepsis.

The findings on heparin therapy in septic patients have generated many controversies in clinical literature. Several studies and meta-analyses support the administration of heparin as safe and has been associated with decreased mortality in sepstic patients [74–77]. However, Yamakawa et al. found that anticoagulant treatment is associated with reduced mortality only in subgroups of patients with sepsis-induced coagulopathy and/or those who were very severely ill [78]. Current research on therapeutic anticoagulation in patients with COVID-19 shows that prophylactically administered therapeutically dosed heparin does not improve the critically ill patients’ outcome or mortality rate. In fact, the studies found it could be harmful [79]. In contrast, in patients with moderate COVID-19, therapeutic anticoagulation may reduce the need for organ support [79]. Another randomized clinical trial found prophylactically administered therapeutic-dose anticoagulation reduced death compared with institutional standard thromboprophylaxis only among patients with COVID-19 with extremely elevated D-dimer levels [80].

The results obtained herein suggest that heparin therapy improves prognosis in patients with abnormal coagulation, but prolonged PTT due to excessive anticoagulation and bleeding complications should be avoided. These results may guide futures studies looking at which patients may benefit from therapeutic anticoagulation. One of the main concerns is the potential risk of major hemorrhage. Besides bleeding, the main adverse effect of heparin is heparin-induced thrombocytopenia (HIT). Furthermore, as most septic patients had hypoperfusion, the subcutaneous route is less suitable due to poor absorption. This might have attenuated the efficacy of heparin because of poor bioavailability.

A 7-layer CNN and a DCQMFF model were presented for the survival prediction of sepsis patients based on indicators obtained from routine blood tests. The ReLU function applied in the CNN model mitigates the gradient vanishing problem when optimizing the deep neural network. A set of methods has been generated as baseline survival prediction tools. The SOFA score has been widely validated across healthcare settings and environments. Compared with other promising ML algorithms including random forest, logistic regression, and LASSO methods, the proposed models show better performance in terms of accuracy, precision (random forest as an exception), recall and AUC for the test datasets. Especially, both the DCQMFF and CNN based models performed well in the verification set from MIMIC-IV (Table 3).

The DCQMFF model was proposed to incorporate the relationship between 11 features of sepsis patients into the prediction system and predict the patients’ 28-day survival rate. These 11 features were closely related to the patients’ survival state. DCQMFF enables obtaining the patients’ survival probability using a comprehensive weighted value of 11 features. An application platform based on DCQMFF was established to quickly predict the 28-day survival rate. Combining the prediction results with clinical experience, physicians can stratify septic patients into risk categories, which can guide management and discussions surrounding prognosis.

SOFA score is a mortality prediction tool based on six organ systems and has been widely validated as a tool for assessment of the acute morbidity across healthcare settings and environments. However, SOFA score is not a specific tool for predicting prognosis of sepsis [29]. CNN model is capable to learn the internal laws and representation levels of sample data automatically, and it is purposed of learning the mapping relationship between sample data and corresponding class labels of the data. By using CNN model, the survival rate prediction accuracy reached 92% in the current work. To establish an APP, we chose 11 features reflecting the patient's physical characteristics to feed DCQMFF model. DCQMFF is an improved quadratic fitting function. To further solve the nonlinear problems, we applied a double coefficient quadratic multivariate fitting function.

Previous studies have shown that traditional machine learning methods have high requirements for input features. For high noise data, over-fitting phenomenon is prone to occur in random forest model. For nonlinear data, the logistic regression model shows worse experimental performance. To solve nonlinear problems, SVM model needs to choose kernel function carefully. For high-dimensional sparse feature data, Gradient boosting model is unsuitable. And if the input features are dependent and relevant, Bayesian model will barely be a good choice. To sum up, traditional ML methods have strict requirements for the input data, which requires a manual and careful selection of input features. Therefore, CNN and DCQMFF perform better than traditional ML.

To provide additional insight into the effectiveness of CNN and DCQMFF within each phenotype (generated by K-means clustering), we applied the two classifiers on each cluster (phenotype) individually. The results were shown in Additional file 1: Table S2. Due to the data imbalance, especially in C_3, only 65 cases (46 in training set and 19 in test set) assigned, under-fitting or over-fitting occurred in the performance. As we know deep learning works poor with small-sized data set, larger prediction error would be expected with respect to the small amount of data in this case. For example, in both the test and verification set of C_3, the performance of both CNN and DCQMFF was not satisfactory.

Using the proposed deep-learning methods, the death risk of sepsis patients can be accurately predicted using routine blood tests. The DCQMFF model can help optimize medical resources and eliminate the need to conduct additional tests, thereby reducing the associated risks. The model can be implemented in medical institutions of different levels. However, although the considered models are promising, they are limited by their retrospective nature. Prospective cohort studies are needed to validate their effectiveness further.

## Conclusion

The K-means clustering model successfully identified the distinct sepsis phenotypes associated with survival, and significant features correlated with mortality were identified. The findings suggest that sepsis patients with abnormal coagulation had poor outcomes. The anticoagulation effects of appropriate heparin sodium treatment may improve organ failure caused by extensive micro thrombosis. The proposed CNN and DCQMFF models performed well in predicting the survival rate of sepsis patients. Furthermore, the DCQMFF-based application platform is generated to fast and accurately predict the 28-day survival rate using only 11 blood test variables from patients. In the future, prospective cohort studies will be conducted to validate the proposed models’ effectiveness further.

## Supplementary Information

**Table S1.** The features of 2371 cases according to blood analysis. **Table S2.** The performance of CNN and DCQMFF in different phenotypes. **Figure S1.** Other features of each cluster. **A** Alanine Aminotransferase (ALT, IU/L), **B**. Albumin (g/dL), **C**. Alkaline Phosphatase (log (IU/L)), **D**. Anion Gap (mEq/L), **E**. Aspirate Aminotransferase (log (IU/L)), **F**. Basophils (%), **G**. Bicarbonate (mEq/L), **H**. Bilirubin (IU/L), **I**. Calcium (mg/dL), **J**. Chloride (mEq/L), **K**. Creatinine (mg/dL), **L**. Eosinophils (%), **M**. Glucose (mg/dL), **N**. Hematocrit (%), **O**. Hemoglobin (g/dL). **Figure S2** Other futures of each cluster. **A**. INR(PT), **B**. Lactate (mmol/L), **C**. Magnesium (mg/dL), **D**. MCH (pg), **E**. MCHC (%), **F**. MCV (fL), **G**. Monocytes (%), **H**. pH (units), **I**. Phosphate (mg/dL), **J**. Platelet Count (log (K/uL)), **K**. Potassium (mEq/L), **L**. PT (sec), M. RDW (%), **N**. Red Blood Cells (m/uL), **O**. Sodium (mEq/L), **P**. Urea Nitrogen (log (mg/dL)). **Figure S3** Survival nomograms and its’ calibration curve of each cluster. Survival nomogram and prediction carve for C_1 (A1 and A2:), C_2 (B1 and B2), C_3 (C1 and C2), C_4 (D1 and D2), respectively. In the calibration plot, Nomogram-predicted 28-day survival rates are on the x-axis, actual survival rates are plotted on the y-axis. The gray line represents the ideal fit where the nomogram-predicted probability matches the actual probability.

## Data Availability

The datasets presented in this study can be found in MIMIC-III (https://doi.org/10.13026/C2XW26) and MIMIC-IV (https://doi.org/10.13026/a3wn-hq05) datasets.

## Abbreviations

MIMI*C*: Medical information mart for intensive care
CNN: Convolutional neural network
DCQMFF: Double coefficient quadratic multivariate fitting function
C_1: First cluster
C_2: Two cluster
C_3: Three cluster
C_4: Four cluster
SOFA: Sequential organ failure assessment
qSOFA: Quick sequential organ failure assessment
PTT: Partial thromboplastin time
PT: Prothrombin time
SIC: Septic coagulation disease score
APACHE II: Acute physiology and chronic health evaluation II
SIRS: Systemic inflammatory response syndrome
ML: Machine learning
SVM: Support vector machine
ICU: Intensive care unit
T-test: Student’s t test
F-Test: Joint hypotheses test
ANOVA: One-way analysis of variance
ReLU: Rectified linear unit
INR-PT: International standardized ratio of prothrombin time
pH: Hydrogen ion concentration
ROC: Receiver operating characteristic curve
TPR: True positive rate
FPR: False positive rate
AUC: The area under the ROC curve
PCA: Principal component analysis
GCS: Glasgow coma scale
PaO2: Alveolar oxygen partial pressure
FiO2: Fraction of inspiration O2
LASSO: Least absolute shrinkage and selection operator
HIT: Heparin-induced thrombocytopenia
