# Supplementary material for: Clinical applications of machine learning in the survival prediction and classification of sepsis: coagulation and heparin usage matter
Source: J Transl Med. 2022 Jun 11;20:265. doi: 10.1186/s12967-022-03469-6 (PMC9187899; doi:10.1186/s12967-022-03469-6)
Supplement: Supplementary file 1 — Table S1. The features of 2371 cases according to blood analysis. Table S2. The performance of CNN and DCQMFF in different phenotypes. Figure S1. Other features of each cluster. A Alanine Aminotransferase (ALT, IU/L), B. Albumin (g/dL), C. Alkaline Phosphatase (log (IU/L)), D. Anion Gap (mEq/L), E. Aspirate Aminotransferase (log (IU/L)), F. Basophils (%), G. Bicarbonate (mEq/L), H. Bilirubin (IU/L), I. Calcium (mg/dL), J. Chloride (mEq/L), K. Creatinine (mg/dL), L. Eosinophils (%), M. Glucose (mg/dL), N. Hematocrit (%), O. Hemoglobin (g/dL). Figure S2 Other futures of each cluster. A. INR(PT), B. Lactate (mmol/L), C. Magnesium (mg/dL), D. MCH (pg), E. MCHC (%), F. MCV (fL), G. Monocytes (%), H. pH (units), I. Phosphate (mg/dL), J. Platelet Count (log (K/uL)), K. Potassium (mEq/L), L. PT (sec), M. RDW (%), N. Red Blood Cells (m/uL), O. Sodium (mEq/L), P. Urea Nitrogen (log (mg/dL)). Figure S3 Survival nomograms and its’ calibration curve of each cluster. Survival nomogram and prediction carve for C_1 (A1 and A2:), C_2 (B1 and B2), C_3 (C1 and C2), C_4 (D1 and D2), respectively. In the calibration plot, Nomogram-predicted 28-day survival rates are on the x-axis, actual survival rates are plotted on the y-axis. The gray line represents the ideal fit where the nomogram-predicted probability matches the actual probability. [file 12967_2022_3469_MOESM1_ESM.docx]

Clinical Applications of Machine Learning in the Survival Prediction and Classification of Sepsis: Coagulation and Heparin Usage Matter

Fei Guo^1†^, Xishun Zhu^2†^, Zhiheng Wu^3^, Li Zhu ^3^, Jianhua Wu ^3*^, Fan Zhang^4*^

^1^Ningbo Institute for Medicine & Biomedical Engineering Combined Innovation, Ningbo Medical Treatment Centre Lihuili Hospital, Ningbo University, Ningbo, Zhejiang 315040, China

^2^School of Mechatronics Engineering, Nanchang University, Nanchang 330031, China

^3^School of Information Engineering, Nanchang University, Nanchang 330031, China

^4^Department of Critical Care Medicine, Qilu Hospital, Cheeloo College of Medicine, Shandong University, Jinan 250012, China

† These authors contribute to the work equally and are regarded as co-first authors

***Correspondence:**

zhfansdu@163.com; [jhwu@ncu.edu.cn](mailto:jhwu@ncu.edu.cn)

# Supplemental Data

**FIGURE S1** Other features of each cluster. A. Alanine Aminotransferase (ALT, IU/L), B. Albumin (g/dL), C. Alkaline Phosphatase (log (IU/L)), D. Anion Gap (mEq/L), E. Aspirate Aminotransferase (log (IU/L)), F. Basophils (%), G. Bicarbonate (mEq/L), H. Bilirubin (IU/L), I. Calcium (mg/dL), J. Chloride (mEq/L), K. Creatinine (mg/dL), L. Eosinophils (%), M. Glucose (mg/dL), N. Hematocrit (%), O. Hemoglobin (g/dL).

**FIGURE S2|** Other futures of each cluster. A. INR(PT), B. Lactate (mmol/L), C. Magnesium (mg/dL), D. MCH (pg), E. MCHC (%), F. MCV (fL), G. Monocytes (%), H. pH (units), I. Phosphate (mg/dL), J. Platelet Count (log (K/uL)), K. Potassium (mEq/L), L. PT (sec), M. RDW (%), N. Red Blood Cells (m/uL), O. Sodium (mEq/L), P. Urea Nitrogen (log (mg/dL)).

**FIGURE S3**| Survival nomograms and its’ calibration curve of each cluster. Survival nomogram and prediction carve for C_1 (A1 and A2:), C_2 (B1 and B2), C_3 (C1 and C2), C_4 (D1 and D2), respectively. In the calibration plot, Nomogram-predicted 28-day survival rates are on the x-axis, actual survival rates are plotted on the y-axis. The gray line represents the ideal fit where the nomogram-predicted probability matches the actual probability.

**Table S1 The features of 2371 cases according to blood analysis**

| Features | Survival | Death | *P* value |
| --- | --- | --- | --- |
| General features and blood analysis |  |  |  |
| number of patients (percentage) | 1891 (69.6%) | 480 (30.4%) |  |
| Age (years): average, median, min, max | 66, 67, 19, 89 | 68,72, 27, 89 |  |
| Male, Num (percentage) | 635 (33.6%) | 287 (59.8%) | 0.000 |
| Lactate, mmol/L, median (IQR) | 2.2 (1.5–3.3) | 2.9 (1.8–5) | 0.000 |
| pH, units, median (IQR) | 7.34 (7.32–7.41) | 7.34 (7.25–7.39) | 0.000 |
| Alanine Aminotransferase (ALT), IU/L, median (IQR) | 25 (15–52) | 27 (15–54) | 0.128 |
| Albumin, g/dL, median (IQR) | 2.9 (2.6–3.4) | 2.7 (2.2–3) | 0.000 |
| Alkaline Phosphatase, IU/L, median (IQR) | 107 (75–151) | 124.5 (85–197.25) | 0.000 |
| Anion Gap, mEq/L, median (IQR) | 16 (14–20) | 18 (15–21) | 0.000 |
| Aspirate Aminotransferase (AST), IU/L, median (IQR) | 33 (21–68) | 41(24–92) | 0.000 |
| Bicarbonate, mEq/L, median (IQR) | 23 (20–27) | 22 (18–26) | 0.000 |
| Bilirubin, Total, IU/L, median (IQR) | 0.6 (0.3–1.1) | 0.7 (0.4–1.5) | 0.000 |
| Calcium, Total, mg/dL, median (IQR) | 8.3 (7.7–9) | 8 (7.4–8.8) | 0.000 |
| Chloride, mEq/L, median (IQR) | 101(97–106) | 102 (97–108) | 0.042 |
| Creatinine, mg/dL, median (IQR) | 1.5 (1–2.6) | 1.9 (1.2–3) | 0.000 |
| Glucose, mg/dL, median (IQR) | 129 (103–180) | 128.5 (97.8–174.3) | 0.059 |
| Magnesium, mg/dL, median (IQR) | 1.8 (1.6–2.1) | 1.9 (1.6–2.3) | 0.001 |
| Phosphate, mg/dL, median (IQR) | 3.4 (2.6–4.3) | 4 (3.2–5.3) | 0.000 |
| Potassium, mEq/L, median (IQR) | 4.3 (3.8–4.9) | 4.4 (3.9–5.2) | 0.001 |
| Sodium, mEq/L, median (IQR) | 138 (134–141) | 138 (134–142) | 0.155 |
| Urea Nitrogen, mg/dL, median (IQR) | 29 (19–48) | 40 (26–61.25) | 0.000 |
| Basophils, percentage, median (IQR) | 0.1 (0–0.3) | 0 (0–0.2) | 0.000 |
| Eosinophils, percentage, median (IQR) | 0.4 (0–1.3) | 0.1 (0–0.7) | 0.000 |
| Hematocrit, percentage, median (IQR) | 33.6 (29.9–37.7) | 32.3(28.8–37.9) | 0.016 |
| Hemoglobin, g/dL, median (IQR) | 11 (9.7–12.6) | 10.5(9.275–12.3) | 0.000 |
| INR(PT), NULL, median (IQR) | 1.3 (1.1–1.6) | 1.5 (1.2–2.2) | 0.000 |
| Lymphocytes, percentage, median (IQR) | 8.5 (5–14.5) | 6.05 (3.575–12) | 0.000 |
| MCH, pg, median (IQR) | 30 (28.4–31.7) | 30.2 (28.2–31.7) | 0.799 |
| MCHC, percentage, median (IQR) | 33.1 (32–34.2) | 32.6 (31.3–33.6) | 0.000 |
| MCV, fL, median (IQR) | 91 (86–95) | 92 (86.75–97) | 0.002 |
| Monocytes, percentage, median (IQR) | 3.8 (2.2–5.4) | 3.2 (2–5.15) | 0.056 |
| Neutrophils, percentage, median (IQR) | 81.1 (72.65–88.5) | 82.05 (71.0–89.4) | 0.541 |
| Platelet Count, K/μL, median (IQR) | 232 (156.5–333) | 215.5 (133.8–328) | 0.031 |
| PT, sec, median (IQR) | 14.4 (13.2–17.2) | 15.6 (13.8–20.76) | 0.000 |
| PTT, sec, median (IQR) | 30.7 (26.7–36.7) | 33.7 (28.2–42.13) | 0.000 |
| RDW, percentage, median (IQR) | 15.4 (14.2–17) | 16.4 (14.9–18.2) | 0.000 |
| Red Blood Cells, m/μL, median (IQR) | 3.72 (3.26–4.21) | 3.58 (3.09–4.1) | 0.000 |
| White Blood Cells, K/μL, median (IQR) | 11.7 (7.7–16.95) | 13.8 (8.7–20.13) | 0.000 |

**TABLE S2** The performance of CNN and DCQMFF in different phenotypes.

| **Methods** | | | **Accuracy** | **Precision** | **Recall** | ***F*1** | **AUC** |
| --- | --- | --- | --- | --- | --- | --- | --- |
| DCQMFF  (Proposed) | C_1 | training | 0.844 | 0.826 | 0.442 | 0.576 | 0.706 |
|  |  | test | 0.833 | 0.555 | 0.455 | 0.500 | 0.686 |
|  |  | val | 0.783 | 0.500 | 0.308 | 0.381 | 0.611 |
|  | C_2 | training | 0.821 | 0.680 | 0.087 | 0.155 | 0.539 |
|  |  | test | 0.838 | 0.429 | 0.056 | 0.098 | 0.521 |
|  |  | val | 0.815 | 0.500 | 0.048 | 0.086 | 0.518 |
|  | C_3 | training | 0.600 | 0.200 | 0.333 | 0.250 | 0.500 |
|  |  | test | 0.700 | 0.000 | 0.000 | 0.000 | NaN |
|  |  | val | 0.800 | 0.000 | 0.000 | 0.000 | NaN |
|  | C_4 | training | 0.856 | 0.906 | 0.592 | 0.716 | 0.782 |
|  |  | test | 0.700 | 0.412 | 0.583 | 0.483 | 0.660 |
|  |  | val | 0.740 | 0.571 | 0.533 | 0.552 | 0.681 |
| CNN(Proposed) | C_1 | training | 0.835 | 0.636 | 0.400 | 0.491 | 0.806 |
|  |  | test | 0.714 | 0.444 | 0.266 | 0.333 | 0.621 |
|  |  | val | 0.696 | 0.455 | 0.313 | 0.370 | 0.645 |
|  | C_2 | training | 0.905 | 0.896 | 0.566 | 0.693 | 0.933 |
|  |  | test | 0.808 | 0.281 | 0.173 | 0.214 | 0.754 |
|  |  | val | 0.802 | 0.429 | 0.238 | 0.306 | 0.771 |
|  | C_3 | training | 0.718 | 0.333 | 0.125 | 0.182 | 0.677 |
|  |  | test | 0.750 | 0.00 | 0.00 | 0.00 | NaN |
|  |  | val | 0.625 | 0.500 | 0.333 | 0.400 | 0.333 |
|  | C_4 | training | 0.913 | 0.943 | 0.733 | 0.825 | 0.954 |
|  |  | test | 0.708 | 0.500 | 0.357 | 0.416 | 0.702 |
|  |  | val | 0.646 | 0.444 | 0.250 | 0.320 | 0.566 |
